# A short protocol using dexamethasone and monophosphoryl lipid A generates tolerogenic dendritic cells that display a potent migratory capacity to lymphoid chemokines

**DOI:** 10.1186/1479-5876-11-128

**Published:** 2013-05-24

**Authors:** Paulina García-González, Rodrigo Morales, Lorena Hoyos, Jaxaira Maggi, Javier Campos, Bárbara Pesce, David Gárate, Milton Larrondo, Rodrigo González, Lilian Soto, Verónica Ramos, Pía Tobar, María Carmen Molina, Karina Pino-Lagos, Diego Catalán, Juan Carlos Aguillón

**Affiliations:** 1 Immune Regulation and Tolerance Research Group, Programa Disciplinario de Inmunología, ICBM, Facultad de Medicina, Universidad de Chile, Santiago, Chile; 2 Millennium Institute on Immunology and Immunotherapy, Santiago, Chile; 3 Banco de Sangre, Hospital Clínico de la Universidad de Chile, Santiago, Chile; 4 Sección de Reumatología, Hospital Clínico de la Universidad de Chile, Santiago, Chile; 5 Laboratorio de Evasión Inmune, Programa Disciplinario de Inmunología, ICBM, Facultad de Medicina, Universidad de Chile, Santiago, Chile

**Keywords:** Tolerance, Monocyte-derived dendritic cells, Chemokine receptors, CCR7, CXCR4, Cell-based therapy

## Abstract

**Background:**

Generation of tolerogenic dendritic cells (TolDCs) for therapy is challenging due to its implications for the design of protocols suitable for clinical applications, which means not only using safe products, but also working at defining specific biomarkers for TolDCs identification, developing shorter DCs differentiation methods and obtaining TolDCs with a stable phenotype. We describe here, a short-term protocol for TolDCs generation, which are characterized in terms of phenotypic markers, cytokines secretion profile, CD4+ T cell-stimulatory ability and migratory capacity.

**Methods:**

TolDCs from healthy donors were generated by modulation with dexamethasone plus monophosphoryl lipid A (MPLA-tDCs). We performed an analysis of MPLA-tDCs in terms of yield, viability, morphology, phenotypic markers, cytokines secretion profile, stability, allogeneic and antigen-specific CD4+ T-cell stimulatory ability and migration capacity.

**Results:**

After a 5-day culture, MPLA-tDCs displayed reduced expression of costimulatory and maturation molecules together to an anti-inflammatory cytokines secretion profile, being able to maintain these tolerogenic features even after the engagement of CD40 by its cognate ligand. In addition, MPLA-tDCs exhibited reduced capabilities to stimulate allogeneic and antigen-specific CD4+ T cell proliferation, and induced an anti-inflammatory cytokine secretion pattern. Among potential tolerogenic markers studied, only TLR-2 was highly expressed in MPLA-tDCs when compared to mature and immature DCs. Remarkable, like mature DCs, MPLA-tDCs displayed a high CCR7 and CXCR4 expression, both chemokine receptors involved in migration to secondary lymphoid organs, and even more, in an *in vitro* assay they exhibited a high migration response towards CCL19 and CXCL12.

**Conclusion:**

We describe a short-term protocol for TolDC generation, which confers them a stable phenotype and migratory capacity to lymphoid chemokines, essential features for TolDCs to be used as therapeutics for autoimmunity and prevention of graft rejection.

## Background

Dendritic cells (DCs) are professional antigen-presenting cells that play a crucial role in antigen-specific immune responses and tolerance induction. DCs normally reside in peripheral tissues, sensing for the presence of either microbes or endogenous danger signals. Upon recognizing these signals, DCs undergo a complex process of maturation, which include changes in morphology, loss of endocytic receptors, upregulation of antigen presenting (MHC class I and II), costimulatory (CD80 and CD86) and functional activator (CD40) molecules, together to increased secretion of cytokines able to polarize T cells [1]. In addition, mature DCs (mDCs) change their expression pattern of chemokine receptors, acquiring the ability to migrate to secondary lymphoid organs where they encounter T cells. It has been largely demonstrated that this trafficking relies on the expression of the chemokine receptor CCR7, which follows chemotactic gradients of CCL19 and CCL21 [2], however, new evidence suggests that this process also requires other chemokine receptor, namely CXCR4 and its ligand CXCL12 [3]. DCs have been also implicated in the pathogenesis of multiple autoimmune diseases, acting as antigen-presenting cells to autoreactive T cells. However, both immature DCs (iDCs) and tolerogenic DCs (TolDCs) are involved in the maintenance of peripheral tolerance. These TolDCs have characteristic features such as a reduced costimulatory capacity and an anti-inflammatory cytokine secretion profile [4], and exert their modulatory activity on autoreactive T cells through several mechanisms such as clonal deletion or anergy [5]. Moreover, TolDCs are able to promote the differentiation and proliferation of T cells with regulatory functions [6].

Current efforts for the treatment of autoimmune diseases and other immune processes requiring tolerance recovery are focused in the development of therapies aimed at inducing antigen-specific immuneregulation, with lesser side effects than wide spectrum immunosuppressive agents [7]. In this sense, TolDCs capable of modulating immune responses in an antigen-specific manner have become a promising therapeutic tool. TolDCs can be generated *ex vivo* from peripheral blood monocytes modulated by different approaches, including conditioning with pharmacological agents such as vitamin D3, dexamethasone (Dex) and rapamycin [8-10]; anti-inflammatory cytokines including IL-10 and TGF-β [5,11]; and genetic modifications such as IL-4 and IL-10 transduction [12,13].

Although numerous investigations have described human TolDCs mechanisms of action *in vitro*, up to date there is only one phase I study describing the administration of TolDCs in type 1 diabetes mellitus patients [14]. Translating laboratory protocols to patients is a challenging step, since several issues related to therapeutic TolDCs generation might be considered, such as i) The use of clinical grade reagents; ii) Cell purity, viability, functionality and stability assurance once they encounter a pro-inflammatory environment, iii) Identification of specific tolerogenic markers for quality control; and iv) Shorter generation times, among others [15].

The present study was aimed at developing a shortened protocol for the generation of human TolDCs modulated with Dex and activated with monophosphoryl lipid A (MPLA), a clinical grade analog for lipopolysaccharide (LPS). A systematic analysis in terms of yield, viability, morphology, phenotypic markers, cytokines secretion profile, stability, allogeneic and antigen-specific CD4+ T-cell stimulatory ability and migration capacity were performed, in order to evaluate the applicability of these cells for clinical trials in autoimmune diseases.

## Methods

### Cell isolation

Buffy coats from healthy volunteers were obtained from the Clinical Hospital’s Blood Bank at Universidad de Chile upon informed consent forms were read and signed. The study was approved by the Clinical Hospital’s Ethical Committee at Universidad de Chile (Santiago, Chile). CD14+ monocytes and CD4+ T cells were obtained by negative selection with RosetteSep® Human Monocyte Enrichment Cocktail and RosetteSep® CD4+ Human T Cell Enrichment Cocktail (StemCell Technologies, Vancouver, BC, Canada), using a density gradient with Ficoll-Paque™ Plus (GE Healthcare Bio-Sciences Corp, Piscataway, NJ, USA).

### Generation of dendritic cells

Monocytes isolated as described above were cultured (2-4×10^6^ cells/ml) for 5 days in AIM-V serum-free therapeutic grade medium (GIBCO BLR, Invitrogen, Grand Island, NY, USA) in the presence of 500 U/mL of 500 U/mL of recombinant human (rh) GM-CSF and 500 U/mL of rhIL-4 (eBioscience, San Diego, CA, USA), refreshing with fresh medium and cytokines on day 3. In order to induce a tolerogenic profile, Dex (1 μM, Sigma-Aldrich, St. Louis, CO, USA) was added on day 3, 48 hours before cell harvest, generating tDCs. The current Good Manufacture Practices (cGMP)-grade MPLA (1 μg/ml, Avanti Polar Lipids Inc, Alabaster, AL, USA) was added on day 4 to obtain mDCs or activated tDCs (MPLA-tDCs). Untreated cells were used as iDCs controls. On day 5, cells were harvested and washed twice with PBS, and phenotypic and functional analyses were performed.

### Flow cytometry analysis

The following antibodies were used for cell surface molecules expression analysis: CD11c APC, CD80 FITC, CD83 FITC, CD40 PE, CD86 PE, MHC II (HLA-DR) FITC, MHC I (HLA-ABC) FITC, CD1a FITC, CD14 FITC, ILT3 PE, PD-L1 PE, TLR-2 PE, CCR5 PE, CCR7 PE, CXCR1 FITC, CXCR2 PE, CXCR4 PE, CD4 PE, CD4 PeCy7, IFNγ APC, all from eBioscience; CCR2 PerCP-Cy5.5 from BioLegend (San Diego, CA, USA) and CCR1 PE from R&D Systems (Minneapolis, MN, USA). Cells were resuspended in PBS + 10% FBS and incubated with antibodies for 45 minutes, then washed twice with PBS + 3% FBS and fixed with IC Fixation Buffer (eBioscience). GILZ PE antibody (eBioscience) was used for intracellular staining. Cells were fixed, resuspended in Permeabilization Buffer (eBioscience) and incubated with the antibody for 20 minutes, then washed twice with Permeabilization Buffer and resuspended in flow cytometry buffer for data collection. For IFNγ intracellular detection cells were stimulated with phorbol-12-myristate-13-acetate (PMA) (Sigma-Aldrich) (50 ng/ml) / ionomycin (Sigma-Aldrich) (1 μg/ml) and treated with brefeldin A (eBioscience) (1 μl/ml) 5 hours prior to cell staining. Data were collected on a FACSCalibur (Beckton Dickinson, San Diego, CA, USA), and analyzed using WinMDI 2.9 software. Cell death was measured by Annexin V and 7-AAD staining (eBioscience).

### Cytokine production assessment

DCs were harvested on day 5 of culture, washed and seeded in 96-well U-bottom plates (BD Biosciences, San Jose, California, USA) at a concentration of 1×10^5^ cells/100 μl/well in AIM-V medium with or without a stable human CD40L-expressing NIH-3 T3 murine fibroblast cell line at a 1:1 ratio for 24 hours. Supernatants were collected and stored at -80°C and later analyzed for cytokine quantification through ELISA sandwich assays using anti-IL-10, anti-IL-12, anti-IL-23, anti-TNFα and anti-TGFβ1 antibodies (eBioscience).

### Allostimulatory assay

To assess alloproliferation, CD4+ T cells from healthy subjects were purified as described above, and labeled with carboxyfluorescein diacetate succinimidyl ester (CFSE) (Sigma-Aldrich) at a concentration of 5 μM for 40×10^6^ cells/ml. Allogeneic DCs were washed three times and seeded in 96-well U-bottom plates (BD Biosciences) (2×10^5^/200 μl) with CFSE marked CD4+ T cells at a 1:2 DC/T cell ratio for 6 days in RPMI medium supplemented with 10% heat-inactivated fetal calf serum at 37°C and 5% CO_2_. Anti-human CD3 antibody-coated wells (eBiocience) were used to stimulate CD4+ T cells as a positive control. CFSE fluorescence dilution on CD4+ T cells was analyzed by flow cytometry as a measure of cell proliferation.

### Antigen-specific proliferation assay

On day 4 of DC generation, tuberculin purified protein derivative (PPD) (Staten Serum Institute, Copenhagen, Denmark) (10 μg/ml) or no antigen was added to cells, which after 4 hours were stimulated with MPLA (1 μg/ml) to obtain MPLA-tDCs and mDCs. tDCs received Dex as unique stimulus while iDCs did not receive any stimulation. On day 5, (8×10^4^/200 μl) DCs loaded or not with PPD were co-cultured with autologous CD4+ T cells labeled with CFSE at a 1:2 DC/T cell ratio for 6 days. Cell proliferation was determined by CFSE fluorescence dilution on CD4+ T cells by flow cytometry.

### Chemotaxis assays

DCs migration was assessed *in vitro* by using a transwell system (5 μm pore size polycarbonate membrane inserts, Corning Costar, NY, USA). DCs (1.5×10^5^) were seeded in the upper chamber, and AIM-V medium alone or with 250 ng/ml of the chemokines RANTES/CCL5, SDF-1α/CXCL12 or MIP-3β/CCL19, all from Peprotech (Rocky Hill, NJ, USA), were added in the lower chamber. DCs migration was analyzed after a 4-hour incubation period at 37°C and 5% CO_2_ by counting DCs on lower chamber using flow cytometry. DC migration is expressed as a migration index, which is the result of the ratio between the cells migrating towards specific chemokines and cells migrating towards medium alone.

### Statistical analyses

One-way ANOVA for repeated measures and Tukey post-tests analyses were done using Prism 5.01 Graphpad Software (San Diego, CA, USA). For chemotaxis assays, paired t-tests were used for comparisons between different DC conditions.

## Results

### Monocytes differentiate to MPLA-tDCs after a 5-day culture protocol

**Figure 1:**
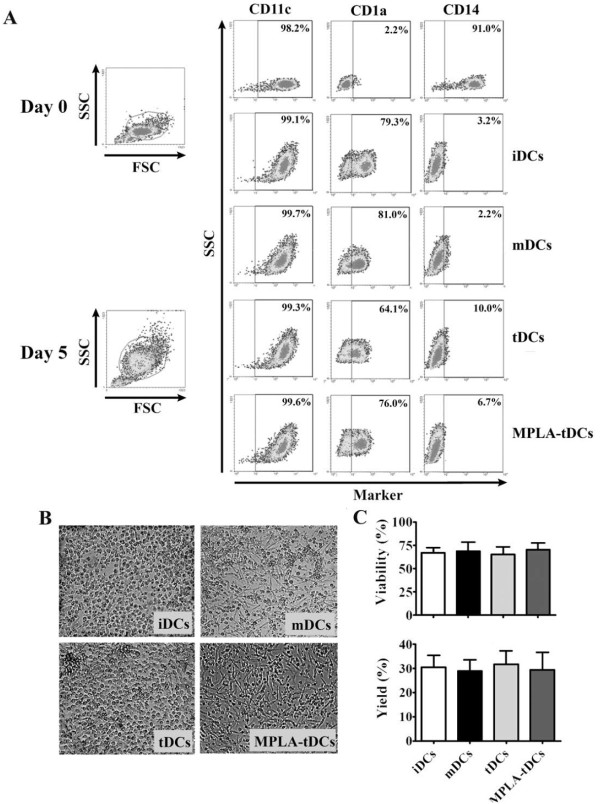
**In a 5-day culture human monocytes differentiate into dendritic cells (DCs) according to lineage markers and cellular morphology.** After 5 days incubation DCs are CD11c + CD1a + and show lower expression of CD14 compared to monocytes. They also exhibit a typical DC morphology. (**A**) 5-day monocyte-derived DCs were differentiated under multiple stimulatory conditions: DC maturated with MPLA (mDCs), DCs conditioned with dexamethasone (tDCs), tDCs activated with MPLA (MPLA-tDCs) or immature DCs left untreated (iDCs). Cells were stained with CD14, CD11c and CD1a to assess their development towards fully differentiated DCs. Density plots of monocytes (day 0) and DCs (day 5) after each differentiation scheme are shown. Plots are representative of 5 independent experiments. (**B**) Cell morphology was evaluated by light microscopy on each DCs group on day 5. Representative microphotographs are shown. (**C**) Cell viability percentage on day 5 is expressed as the percentage of Annexin V and 7-AAD negative cells (mean ± SEM), while DC yield is expressed as a percentage of DCs obtained on day 5 related to the initial number of monocytes cultured per condition (mean ± SEM) (n = 7).

Our main goal was the development of a short-term protocol for TolDC generation for using in clinical applications. MPLA-tDCs were generated from human monocytes through a 5-day protocol instead of the standard 7-day culture duration [4,16,17], using Dex as tolerizing agent and MPLA as a replacement of LPS for DC activation, avoiding the toxicity displayed by the last one. tDCs, iDCs and mDCs were also differentiated under the same 5-day protocol and they were used as controls as described in Materials and Methods. To assess the monocyte differentiation into DCs, the expression of CD11c, CD1a and CD14 cellular membrane markers was evaluated on days 0 and 5. Phenotypic analyses revealed that on day 0, monocytes expressed high levels of their phenotypic marker CD14 and also expressed CD11c, a marker shared with DCs, however they did not express the human DC marker CD1a (Figure 1A). On day 5, all DC groups treated under different schemes showed high levels of CD11c and CD1a expression, together with a loss of CD14 expression (Figure 1A).

When cellular morphology of 5-day generated DCs was examined, tDCs exhibited a round shape and they were

**Figure 2:**
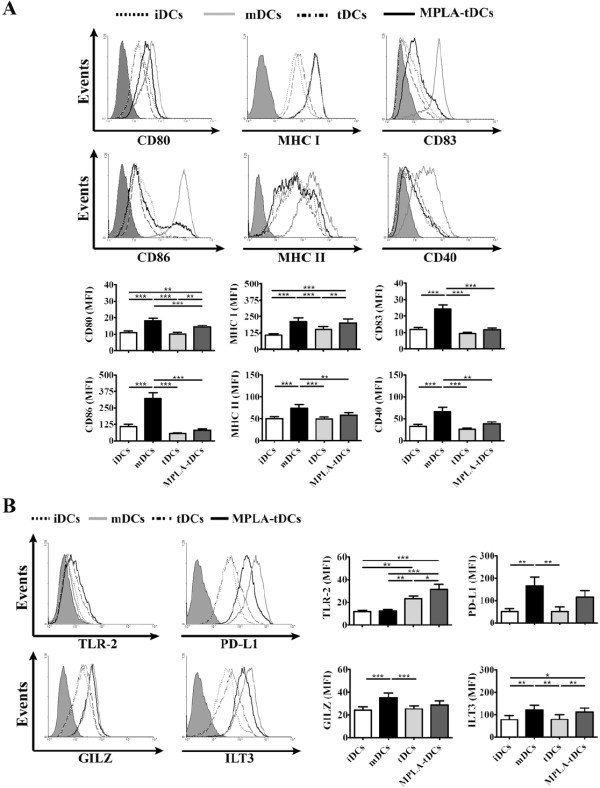
**MPLA-tDCs exhibit a semi-mature surface phenotype and differentially express high levels of TLR-2.** DCs generated following a 5-day protocol were treated with dexamethasone 48 hours before cell harvest for tolerance induction (tDCs). MPLA was added 24 hours before cell collection to obtain mature DC (mDCs) and also to activate tDCs (MPLA-tDCs). Untreated DCs were used as immature controls (iDCs). (**A**) After harvesting, DC surface expression levels of costimulatory (CD80 and CD86), antigen presentation (MHC class I and II), maturation (CD83) and functional activator (CD40) molecules were assessed by flow cytometry, which are shown as representative histograms of each marker (top) and graphic analyses of MFI measurements determined for every marker and expressed as mean ± SEM (bottom) (n = 33). (**B**) TLR-2, PD-L1, GILZ and ILT3 expression on each DC condition was analyzed by flow cytometry to seek for specific tolerogenic DC markers. Representative histograms (left) and graphic analyses for each molecule assessed from 13 independent experiments are shown and represented as mean ± SEM (* P < 0.05; ** P < 0.01; *** P < 0.001).

 strongly attached to culture plates, similarly to iDCs. Unlikely, MPLA-tDCs and mDCs showed a more elongated form and were easily detached by pipetting (Figure 1B). Regarding DC yield and cellular viability, no significant variations between all differentiation conditions were detected (Figure 1C).

### MPLA-tDCs phenotypic analyses revealed an intermediate expression level of functional cellular markers and a high TLR-2 expression

In order to obtain a complete phenotypic characterization for DCs differentiated with various stimuli, the expression of costimulatory (CD80 and CD86), antigen presentation (MHC class I and II), maturation (CD83) and functional activator (CD40) molecules was analyzed by flow cytometry (Figure 2A). Interestingly, both MPLA-tDCs and tDCs displayed low levels of CD80 and CD86 expression compared to mDCs (p < 0.001). In parallel, MPLA-tDCs showed higher expression levels of CD80 than tDCs (p < 0.01). Although MPLA-tDCs and tDCs showed a similar CD86 expression to those of iDCs, MPLA-tDCs showed higher CD80 expression than iDCs (p < 0.01). In addition, MPLA-tDCs displayed higher MHC class I expression than iDCs (p < 0.001) and tDCs (p < 0.01) but similar to that of mDCs. However, for MHC class II, MPLA-tDCs showed expression levels similar to iDCs and tDCs but lower than mDCs (p < 0.01). In the same manner, both MPLA-tDCs and tDCs displayed lower CD83 (p < 0.001 in both cases) and CD40 (p < 0.01 and p < 0.001, respectively) expression levels than mDCs, and a similar expression of both molecules as iDCs and between each other. Taken together, this information suggests that the cellular markers pattern exhibited by tDCs corresponds to an immature stage of phenotypic differentiation, while those displayed by MPLA-tDCs are rather concordant to a transition between immature and mature stages. A similar outcome was seen when activating with LPS (data not shown).

Another important point to be considered in the establishment of protocols for DC generation in order to translate them from the laboratory to the clinic is the identification of specific tolerance molecules to be used as quality control markers. For this purpose, we evaluated the expression of TLR-2, glucocorticoid induced leucine zipper protein (GILZ), the programmed death ligand 1 (PD-L1) and immunoglobulin like transcript (ILT) 3 (Figure 2B), which have been postulated as TolDC markers [18,19]. Of all tolerance markers tested, only TLR-2 was significantly increased in both MPLA-tDCs and tDCs in comparison to iDCs (p < 0.001 and p < 0.01, respectively) and mDCs (p < 0.001 and p < 0.01, respectively). Noteworthy, when tDCs were activated with MPLA they displayed a higher expression level of TLR-2 (p < 0.05) (Figure 2B). We did not observe differences in the expression of GILZ, PD-L1 and ILT3 on MPLA-tDCs or mDCs, but we detected lower levels of these molecules on tDCs in comparison with mDCs (p < 0.001, p < 0.01 and p < 0.01, respectively) (Figure 2B).

### MPLA-tDCs produce low levels of pro-inflammatory cytokines but exhibit a strong IL-10-secreting profile

**Figure 3:**
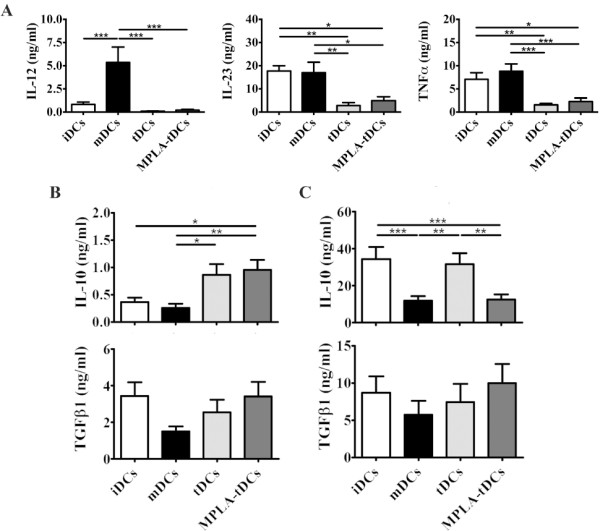
**MPLA-tDCs display an anti-inflammatory cytokine secretion profile with major IL-10 production.** MPLA-tDCs show a pro-tolerogenic cytokine secretion profile with high anti-inflammatory cytokines production and reduced pro-inflammatory cytokines secretion. On day 5, harvested DCs were washed and then incubated for another 24 hours in AIM-V medium alone or co-cultured with the NIH-3 T3 murine fibroblast cell line transfected with human CD40L at a 1:1 DCs:fibroblasts ratio. Culture supernatants were collected and further analyzed by ELISA for pro- and anti-inflammatory cytokines detection. Untreated immature DCs (iDCs) as well as MPLA-matured DCs (mDCs) were used as controls. (**A**) IL-12, IL-23 and TNFα levels determined for all DC groups upon CD40L stimulation. Levels of these cytokines were undetectable in supernatants of DCs without CD40L stimulation. (**B**) IL-10 and TGFβ1 were detected in supernatants of DCs cultured for 24 hours in AIM-V medium. (**C**) IL-10 and TGFβ1 were also determined in supernatants of DCs co-incubated with fibroblasts expressing CD40L. Data in **A**, **B** and **C** correspond to mean ± SEM of 13 independent experiments (* P < 0.05; ** P < 0.01; *** P < 0.001).

The evaluation of pro-inflammatory and anti-inflammatory cytokines secretion patterns allows a more precise characterization of DCs, and also provides important information about the mechanisms through which they could influence immunological processes occurring *in vivo*. As reported by Harry et al. [17], pro-inflammatory cytokines secreted by DCs were undetectable unless stimulating with CD40L-transfected cell lines for 24 hours before supernatants collection. Thus, in the presence of CD40L stimulation, MPLA-tDCs, tDCs and iDCs released significantly lower levels of IL-12 than mDCs (p < 0.001 for all comparisons) (Figure 3A). Likewise, both MPLA-tDCs and tDCs produced lesser IL-23 and TNFα than mDCs (p < 0.05; p < 0.001 for IL-23 and p < 0.01; p < 0.001 for TNFα, respectively) and iDCs (p < 0.05; p < 0.001 for IL-23 and p < 0.01; p < 0.001 for TNFα, respectively).

On the contrary, in the absence of CD40L stimulation, MPLA-tDCs revealed a strong anti-inflammatory profile, secreting significantly more IL-10 than did either iDCs (p < 0.05) or mDCs (p < 0.01) (Figure 3B), while tDCs produced higher IL-10 levels than mDCs (p < 0.05). For TGFβ1, both MPLA-tDCs and tDCs secreted same levels as iDCs and mDCs did (Figure 3B). However, in the presence of CD40L stimulation for 24 hours, MPLA-tDCs, tDCs and iDCs produced almost 12, 40 and 30 times higher amounts of IL-10 than in the absence of CD40L stimulation, respectively (p < 0.001 in all comparisons) (Figure 3C). Interaction with CD40L slightly

 affected TGFβ1 production by DCs, which was almost 3 times more elevated in all conditions studied (Figure 3C).

These results suggest that after tDCs are stimulated with MPLA, cytokine secretion profiles remain unchanged; moreover, when MPLA-tDCs are subjected to a second activation stimulus such as CD40L, they maintain and even strengthen an IL-10-dominated anti-inflammatory profile.

In order to further validate MPLA-tDC stability, we evaluated the expression of costimulatory, maturation and functional activator molecules in differentially stimulated DCs after a strong second activation stimulus with CD40L-transfected cells. The analysis of cellular markers in the presence or absence of CD40L stimulus showed that CD80, CD86, CD83 and CD40 are expressed at similar levels in both conditions (Results not shown). Thus, the phenotypic differences observed between all DC groups studied remained unaltered after CD40 engagement, confirming the stability of MPLA-tDCs.

### MPLA-tDCs modulate allogeneic CD4+ T cells responses

**Figure 4:**
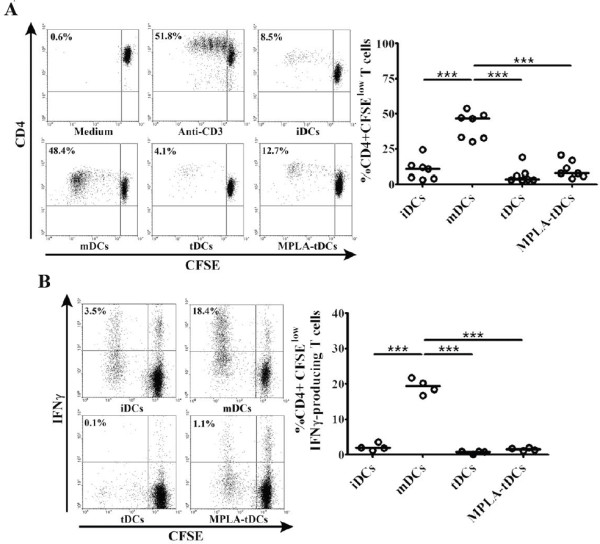
**MPLA-tDCs display reduced capability to stimulate allogeneic CD4+ T cell proliferation and IFNγ production.** MPLA-tDCs and tDCs show decreased allostimulatory capacity compared to MPLA-matured DCs and similar to iDCs. (**A**) Alloproliferative response induced by MPLA-tDCs and tDCs was assessed by co-culturing with allogeneic CD4+ T cells stained with CFSE at a 1:2 DC/T cell ratio for 6 days, and proliferation was determined by CFSE dilution through flow cytometry. CD4+ T cells incubated alone or treated with anti-CD3 (OKT3 clone) were used as controls. Representative flow cytometry plots (left) and graphic analysis of DC allostimulatory capacity for all conditions expressed as percentage of CD4+ CFSElow T cells from 7 independent experiments (right) are shown. (**B**) Production of IFNγ by allogeneic CD4+ T cells was measured on day 6 of culture through intracellular staining after stimulation of cells with PMA/ionomycin for 5 hours and analyzed by flow cytometry. Representative flow cytometry plots (left) and graphic analysis of percentage of IFNγ producing CD4+ CFSElow T cells from 4 independent experiments (right) are shown. Horizontal lines represent median values (*** P < 0.001).

With the purpose to determine the antigen presenting cell ability of the different DC types, we evaluated their ability to induce T cell proliferation in co-cultures with allogeneic CD4+ T lymphocytes. As shown in Figure 4A, MPLA-tDCs, tDCs and iDCs showed reduced capacity to induce T cell alloproliferation when compared to mDCs (p < 0.001 for all 3 conditions). The reduced proliferation of CD4+ T cells achieved by MPLA-tDCs was not caused by the induction of apoptosis, as determined by annexin V and 7-AAD staining (data not shown). To further characterize the type of alloreactive response induced, we also analized intracellular IFNγ expression by CD4+ T cells following co-cultures with all DC stages. As expected, when MPLA-tDCs, tDCs or iDCs were used as stimulators, the percentage of actively proliferating IFNγ-producing CD4+ T cells was significantly decreased compared to that induced by mDCs (p < 0.001 for all comparisons) (Figure 4B).

### MPLA-tDCs induce weak antigen-specific CD4+ T cell proliferation

**Figure 5:**
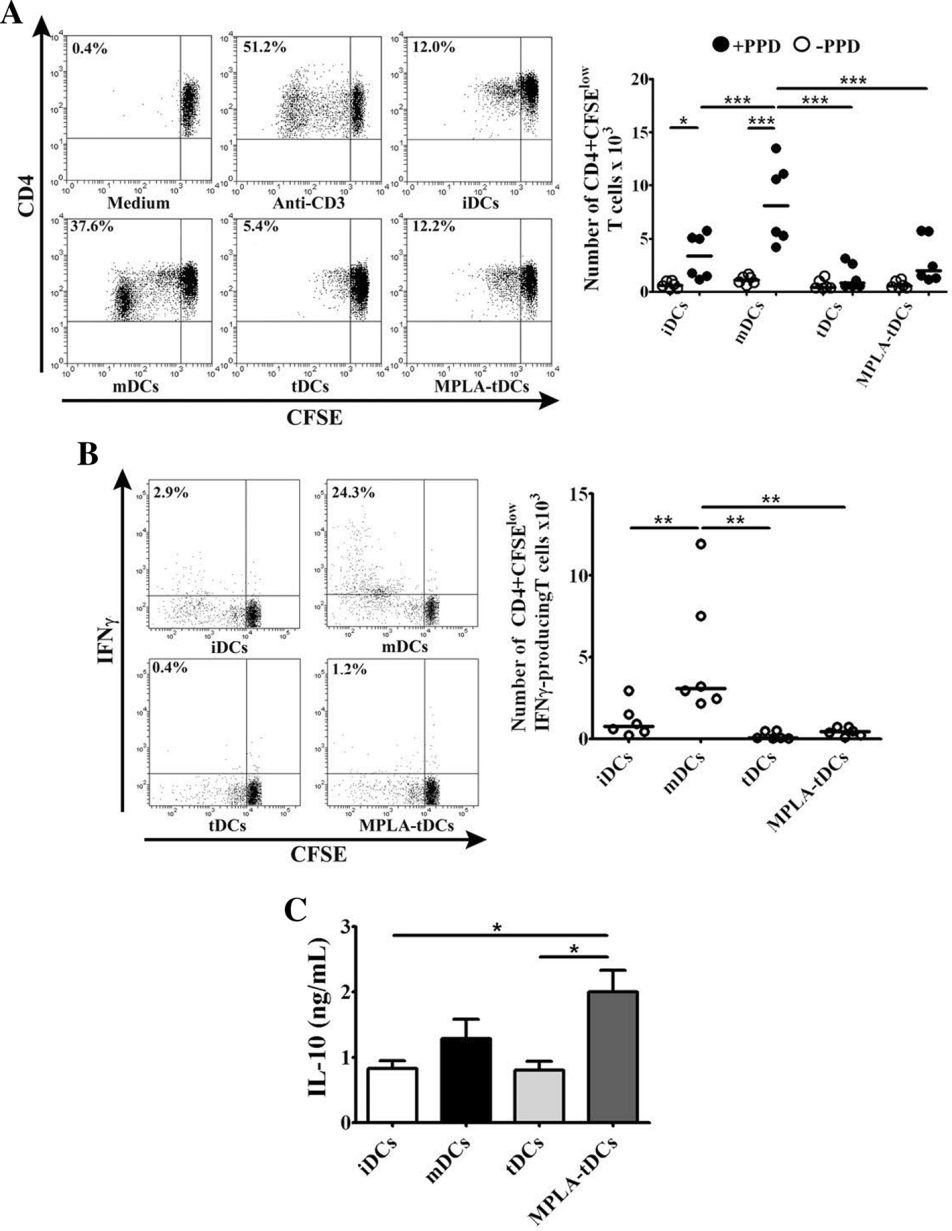
**MPLA-tDCs modulate CD4+ T cell responses in an antigen-specific manner.** CD4+ T cells co-cultured with autologous PPD-loaded MPLA-tDCs exhibited impaired proliferative response together to a reduced IFNγ production and high IL-10 secretion. (**A**) Antigen-specific T cell responses were assessed by co-culturing DCs with autologous CD4+ T cells stained with CFSE at a 1:2 DC/T cell ratio for 6 days, and proliferation was determined by CFSE dilution by flow cytometry. CD4+ T cells incubated alone and CD4+ T cells treated with anti-CD3 (OKT) were used as controls. Representative flow cytometry plots (left) and graphic analysis of DC antigen-specific stimulatory ability for all conditions expressed as number of CD4+ CFSElow T cells for 6 independent experiments (right) are shown. (**B**) Production of IFNγ by autologous T cells was measured on day 6 of culture through intracellular staining after stimulation of cells with PMA/ionomycin for 5 hours and analyzed by flow cytometry. Representative flow cytometry plots (right) and graphic analysis of the number of IFNγ producing CD4+ CFSElow T cells from 6 independent experiments are shown. In **A** and **B** horizontal lines represent median values (* P < 0.05; ** P < 0.01; *** P < 0.001). (**C**) IL-10 secretion levels from DC/T cell culture supernatants were measured using ELISA. Results are represented as the mean ± SEM of 5 independent experiments (* P < 0.05).

We next investigated whether the MPLA-tDC-induced low CD4+ T cell proliferation observed in allogeneic cultures was reproduced in an antigen-specific assay, by co-culturing PPD-loaded DCs with autologous CD4+ T cells stained with CFSE. Unloaded DCs were also co-cultured with T cells as controls. As shown in Figure 5A, antigen-specific CD4+ T cell proliferation response was significantly reduced by PPD-loaded MPLA-tDCs, analogously to tDCs and iDCs, when compared to that displayed by PPD-loaded mDCs (p < 0.001 for all conditions). Furthermore, when CD4+ T cells were co-cultured with MPLA-tDCs, tDCs or iDCs, the percentage of actively proliferating IFNγ-producing CD4+ T cells was significantly reduced compared with that exhibited by mDCs (p < 0.01 for all comparisons) (Figure 5B). In contrast, when IL-10 in culture supernatants was assessed, CD4+ T cells co-cultured with MPLA-tDCs produced higher levels than those secreted by CD4+ T cells cultured with tDCs or iDCs (p < 0.05 for all comparisons) (Figure 5C).

Taken together, all these results suggest that MPLA-tDCs can modulate both allogeneic and antigen-specific CD4+ T cell responses, reducing their proliferation and polarizing their proinflammatory cytokine profile into an anti-inflammatory one.

### Activation of tDCs with MPLA confers them the ability to migrate towards lymphoid tissue-homing chemokines

To exert an effective antigen-specific immunoregulatory response *in vivo*, TolDCs should be capable to migrate to secondary lymphoid tissues where they will present antigens to T cells under a pro-tolerogenic context. However,

 unlike mDCs, Dex-induced TolDCs have been described to express low CCR7 levels, the most relevant lymphoid tissue-homing chemokine receptor [2]. In addition, Dex-induced TolDCs are unable to migrate *in vitro* towards a CCL19 gradient, the specific ligand of CCR7, unless they become activated by LPS or an analog as MPLA [20].

**Figure 6:**
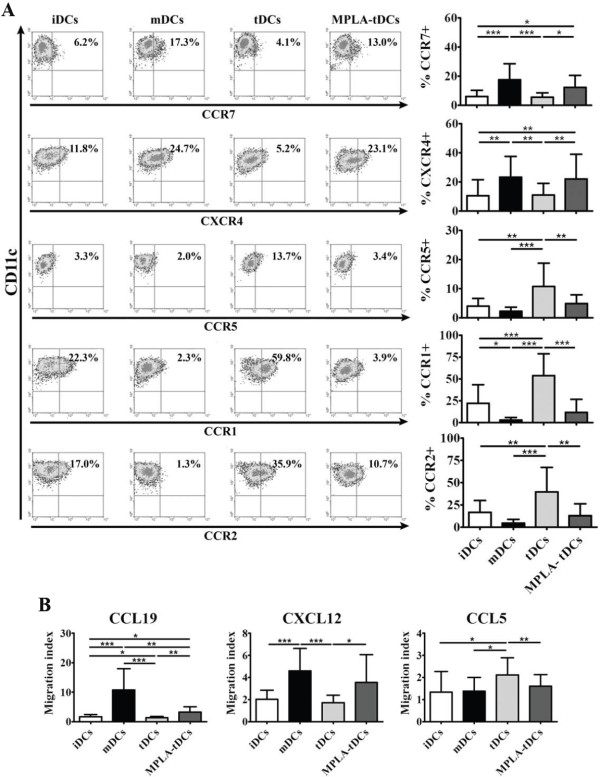
**MPLA-activated tDCs acquire secondary lymphoid homing capacity in response to CCR7 and CXCR4 ligands compared to non-activated tDCs.** MPLA activation of tDCs induces upregulation of migratory receptors CCR7 and CXCR4 and enhances chemotactic responsiveness to their ligands CCL19 and CXCL12. MPLA was added 24 hours before cell harvest to induce tDCs activation, generating MPLA-tDCs. After cell collection on day 5, DCs chemokine receptors expression profile was evaluated by flow cytometry together with their migratory response to relevant chemokines. tDCs without MPLA activation, mature (mDCs) and immature controls (iDCs) were also studied. (**A**) Representative density plots for each chemokine among CD11c + DCs (left). Data are also shown as the mean ± SD percentage of CD11c + DCs expressing the respective receptor analyzed from 10 independent experiments (right). (**B**) DC migratory capacity in response to 250 ng/ml of CCL19, CXCL12 or CCL5, or to medium alone, was assessed through a transwell assay. DC migration for each chemokine is expressed as the mean ± SD of migration index (Migrating DCs towards chemokine/Migrating DCs towards medium) determined in duplicates in 6 independent experiments (* P < 0.05; ** P < 0.01; *** P < 0.001).

As depicted in Figure 6A, MPLA-tDCs and mDCs displayed a higher CCR7 and CXCR4 surface expression, both chemokine receptors involved in migratory response towards secondary lymphoid organs [3], when compared to tDCs (p < 0.05 and p < 0.001 for CCR7; p < 0.01 in both comparisons for CXCR4, respectively) and to iDCs

 (p < 0.05 and p < 0.001 for CCR7; p < 0.01 in both comparisons for CXCR4, respectively). Expression analyses for chemokine receptors associated to migration towards inflamed tissues, such as CCR1, CCR2 and CCR5, revealed a down-regulation of these molecules on MPLA-tDCs compared to tDCs (p < 0.001; p < 0.01 and p < 0.01, respectively) (Figure 6A). Moreover, tDCs showed the highest expression for all 3 chemokine receptors, even more elevated than iDCs, a feature that was completely reverted after activation with MPLA. The evaluation of CXCR1 and CXCR2 expression did not show significant differences between all DC groups studied (data not shown).

In order to define whether the higher CCR7 and CXCR4 and lower CCR5 expression exhibited by MPLA-tDCs have functional relevance, an *in vitro* migration assay was performed using the ligands of these chemokine receptors: CCL19, CXCL12 and CCL5, respectively (Figure 6B). In agreement with the chemokine receptors expression pattern, iDCs and tDCs exhibited a lower migration index in comparison to mDCs in response to CCL19 and CXCL12 (p < 0.001 in both cases –iDCs and tDCs– for CCL19 and CXCL12); however, this tendency was reverted after activation with MPLA (p < 0.01 for CCL19 and p < 0.05 for CXCL12 compared to tDCs) (Figure 6B). In contrast, MPLA-tDCs showed a lower migratory response towards CCL5 than tDCs (p < 0.01), similar to iDCs and mDCs. These results allow us to conclude that 5-day generated tDCs gain lymphoid tissue-homing capacity in response to CCR7 and CXCR4 ligands only after receiving a MPLA activation stimulus.

## Discussion

TolDCs have become a promising tool to be used as therapy in autoimmune diseases and transplantation. The present study reports the development of an alternative protocol for TolDCs generation for clinical purposes, using Dex as immunomodulator and the non-toxic LPS derivative MPLA as TolDCs activator. Dex is known to be an anti-inflammatory and immunosuppressive agent, which has been widely used to treat several autoimmune disorders and prevent graft rejection [21]. In addition to other pharmacological agents, Dex has been extensively studied for TolDCs generation in rodents [22] as well as in humans [23]. An interesting approach has been the generation of “alternatively activated” TolDCs, which after receiving a modulating Dex plus vitamin D3 stimulus are activated with LPS [20,24]. These TolDCs have shown to be a transitory stage between iDCs and mDCs, with a stable phenotype and capable to increase allograft survival and reduce severity of collagen-induced arthritis [25,26]. Meanwhile, MPLA, a non-toxic synthetic LPS analog that retains the ability to activate a variety of cells through TLR-4 signaling, exhibits a potent immune-stimulatory capacity [27]. Even more, it has been shown that LPS and MPLA stimulation display similar bioactivity in the murine macrophage cell line RAW 264.7, inducing equivalent production of nitric oxide, TNFα and IL-6 [28]. Since MPLA has shown to be a safe, well-tolerated and effective enhancer of immune responses in animals, it has been considered useful as adjuvant for several human vaccines [27-29].

In this study, we aimed to develop a shorter DC-generation protocol instead of the standard 7-day protocols [4,16,17]. We addressed the efficacy of the procedure by analyzing specific DC and monocyte markers at the initial and at the final cellular differentiation stages. Interestingly, after 5 days of culture, monocyte-derived cells acquired a characteristic DC phenotype defined by low CD14 and high CD11c and CD1a expression, the latter completely absent in monocytes as established by others [30]. It is noteworthy that tDCs showed a decreased CD1a expression and exhibited moderate levels of CD14, suggesting a lesser extent of differentiation, probably due to Dex effects. Even more, when Dex was added earlier during the protocol, monocytes fail to differentiate (data not shown), which is in agreement with data reported by Woltman et al. [23]. The effect of Dex on DC differentiation was reversed when tDCs were activated with MPLA.

Even though treatment with Dex can affect tDCs differentiation, there were no effects on cell viability or yield of tDCs and MPLA-tDCs when compared to iDCs and mDCs. These results differ from other studies that show reduced DC viability and yield when treated with Dex [4]. Likely, differences seen in our results in comparison with others may be explained by variations in the experimental procedures such as maturation stimuli, Dex and cytokines concentration, together to the protocol duration.

TolDCs should exhibit an immature or semi-mature phenotype characterized by reduced expression of costimulatory (CD80 and CD86), antigen presentation (MHC class I and II), maturation (CD83) and functional activator (CD40) molecules, which could lead to an alternative antigen presentation and costimulation [1,21]. These features together with an anti-inflammatory cytokine secretion profile give TolDCs the ability to establish tolerance through the induction of anergy or deletion of auto-reactive T cells, and also through the promotion of regulatory T cell responses [31]. In our study, MPLA-tDCs exhibited similar expression levels of costimulatory, antigen presentation, maturation and functional activator molecules as tDCs, indicating that even after receiving an activating stimulus, DCs with a tolerogenic rather than a mature phenotype were obtained.

When considering TolDCs for therapy, the rapid and easy identification of specific markers of tolerogenicity is essential for using them as quality control indicators during TolDCs generation. In this sense, very few molecules have been described to be exclusively expressed on TolDCs, and participating in DC regulatory mechanisms. Thus, the immunoglobulin like transcript inhibitory receptor family members, ILT3 and ILT4, have shown to be upregulated in DCs with regulatory properties and have been associated with TolDCs capacity for inducing anergy in T cells [32]; PD-L1 has been reported to be related with tolerance induction [33]; GILZ has been involved in inducing a tolerogenic/regulatory phenotype when upregulated in DCs [34], while TLR-2 has been shown to be upregulated in TolDCs and can also be inducible by glucocorticoids [19,35]. In the present study, only TLR-2 expression was shown to be upregulated in tDCs, but more important, when these DCs were activated with MPLA they increased the expression level of TLR-2, suggesting that can be used as a quality control marker for the generation of MPLA-tDCs.

Cytokines secreted by DCs during T cell priming play an important role in the subsequent differentiation of effector T cells [36]. MPLA-tDCs generated following our 5-day protocol exhibited an anti-inflammatory cytokine secretion profile, characterized by a low production of IL-12, IL-23 and TNFα, and high production of IL-10. These results are consistent with findings reported by others using similar protocols for DC generation [17,37]. Based on results in experimental models reported by our laboratory and other groups, involving IL-10- and TGFβ1-producing DCs in the generation of CD4+CD25+Foxp3+ regulatory T lymphocytes (Tregs) and IL-10-secreting type 1 regulatory T cells (Tr1), we can speculate that MPLA-tDCs could exert a similar regulatory effect *in vivo*, favoring the induction and expansion of different regulatory T cell populations [38-40]. Additionally, it is well known that IL-10 has an inhibitory effect on IL-12 and IL-23 production [41], which could explain the reduced levels detected for these cytokines in supernatants from tDCs and MPLA-tDCs compared to IL-10 levels measured either with or without CD40L stimulation, thus favoring the establishment of their tolerogenic phenotype. These findings are in agreement with those reported by Harry et al., Anderson et al. and Gárate et al., demonstrating a low production of pro-inflammatory cytokines in TolDCs generated with Dex and VitD3, even after CD40 activation [17,20,37]. However, our results for IL-12 and TNFα production by iDCs and mDCs differ from those obtained by Anderson et al. [20], who using LPS to activate DCs and Dex plus vitamin D3 as tolerizing agents, demonstrated a higher IL-12 and TNFα secretion by iDCs than that detected in mDCs. In our opinion, these differences could be explained mainly by variations in the experimental protocols, including reagents, concentration, and protocol duration. The stability of the cytokine secretion profile displayed by MPLA-tDCs become evident after they received the second activation stimulus with CD40L, since pro-inflammatory cytokines remained lower than those showed by mDCs. In contrast, IL-10 and TGFβ1 levels were detected without activation via CD40L and maintained or even augmented after this strong stimulation. In addition to the cytokine secretion profiles provided, DC surface markers expression, evaluated after 24 hours of CD40L stimulation, demonstrated that MPLA-tDCs are able to maintain a semi-mature phenotype after a second activation stimulus, confirming their stable phenotype. These important features are crucial to be considered when using cell-based therapies for tolerance recovery in autoimmunity, given the strong inflammatory environment that TolDCs will encounter when inoculated.

In order to prevent or reverse progression of autoimmune diseases, auto-reactive T cells need to be deleted or suppressed. In this sense, TolDCs ability to inhibit or suppress antigen-specific T cell proliferation is highly desired to induce tolerance. Our study revealed that tDCs exhibited a weak CD4+ T cell allostimulatory capacity, even after activation with MPLA, as compared to mDCs. More important, these CD4+ T cell weak responses induced by MPLA-tDCs and tDCs in allogeneic cultures were reproduced in an antigen-specific fashion using autologous co-cultures, and even more, PPD-loaded MPLA-tDCs shifted the low IFNγ production profile displayed by CD4+ T cells into a robust IL-10 secretion response. These observations are in agreement with other studies demonstrating that Dex-treated DCs exhibit a reduced T cell stimulatory capacity either in allogeneic or antigen-specific co-cultures [23,26,42]. Considering the MPLA-tDC phenotype, characterized by a high IL-10 and low IL-12 production, and a reduced costimulatory and activation machinery, all features related with induction of regulatory T populations, it still remains to be elucidated whether the CD4+ T cells that proliferate in co-cultures with tDCs and MPLA-tDCs correspond to induced Tregs or Tr1 cells [43,44].

As we stated above, TolDCs must maintain their lymph node-homing capacity mainly through CCR7 expression in order to be able to migrate to DC-T cell zones and exert their regulatory effect. Research on semi-mature DCs with tolerogenic properties and activated with LPS after tolerance induction, demonstrated that this feature is essential for TolDC migratory and antigen presentation capacities [20]. Since our goal is to generate TolDCs for clinical purposes, we used MPLA for tDCs activation, replacing the LPS stimulus described for cell activation, and further evaluated MPLA-tDCs chemokine receptors expression and the migratory response to their cognate ligands. Analysis showed that besides CCR7, activated TolDCs also upregulated CXCR4, another chemokine receptor described to be expressed on mature DCs and involved in DC migration to secondary lymphoid organs [45,46]. Expression of both chemokine receptors in MPLA-tDCs as well as in mDCs suggests that these DC types are capable of migrating to DC-T cell contact sites for antigen presentation, data supported by the results obtained in migration assays, demonstrating that mDCs and MPLA-tDCs display migratory abilities in response to CCL19 and CXCL12, ligands of CCR7 and CXCR4, respectively. On the contrary, both mDCs and MPLA-tDCs exhibited a reduced migratory response to CCL5, ligand of CCR5 and CCR1, a chemokine related to migration of leukocytes towards inflamed tissues, while both chemokine receptors were shown to be upregulated on iDCs [47]. The difference observed in MPLA-tDC migratory response towards CCL19 compared to mDCs, could be related to CCR7 expression levels determined for each DC type, with mDCs exhibiting high response towards CCL19, and showing a tendency to a higher expression of CCR7 compared to MPLA-tDCs. These results are concordant with those reported by Anderson et al. [20] using LPS, and with other previous studies on DCs, showing that MPLA stimulation is enough to induce tDCs activation and migration, by triggering a switch in their chemokine receptor expression profile [45,48,49].

## Conclusions

In synthesis, the present study describes a 5-day protocol for TolDCs generation using Dex as immunomodulatory agent, and MPLA, a LPS substitute, as tDCs activator for obtaining clinical grade TolDCs. These MPLA-tDCs exhibited a semi-mature surface phenotype, an anti-inflammatory cytokine secretion profile, reduced allogeneic and antigen-specific CD4+ T cell stimulatory abilities, and a high expression of chemokine receptors involved in lymphoid tissue homing. All these features could validate them to be considered for future clinical studies. n addition, our results support the use of TLR-2 as a suitable MPLA-tDC specific marker applicable for quality control, another desired requirement for clinical application. This knowledge constitutes an important step forward in the road to implement new tools for generating safe and effective TolDCs, which can be used in therapeutic approaches either in autoimmunity or transplantation.

## Abbreviations

7-AAD: 7-Aminoactinomycin D; CFSE: Carboxyfluorescein diacetate succinimidyl ester; cGMP: Current good manufacture practices; DCs: Dendritic cells; Dex: Dexamethasone; GILZ: Glucocorticoid-induced leucine zipper protein; iDCs: Immature dendritic cells; ILT3: Immunoglobulin-like transcript 3; LPS: Lipopolysaccharide; mDCs: Mature dendritic cells; MPLA: Monophosphoryl lipid A; MPLA-tDC: Dexamethasone and monophosphoryl lipid A-induced tolerogenic dendritic cells; PD-L1: Programmed cell death ligand 1; PPD: Tuberculin purified protein derivative; tDCs: Dexamethasone-induced tolerogenic dendritic cells; TolDCs: Tolerogenic dendritic cells

## Competing interests

The authors declare that they have no competing interests.

## Authors’ contributions

JCA and DC participated in the conception and design of the study, analysis and interpretation of data, and manuscript redaction. PG-G, RM, LH, JM, VR and PT participated performing most of the experiments, data acquisition and manuscript preparation. JC, BP, DG, ML, RG, LS, MCM and KP-L collaborated in data acquisition. All authors read and approved the final manuscript.
